# Defining the broader, medium and narrow autism phenotype among parents using the Autism Spectrum Quotient (AQ)

**DOI:** 10.1186/2040-2392-1-10

**Published:** 2010-06-17

**Authors:** Sally Wheelwright, Bonnie Auyeung, Carrie Allison, Simon Baron-Cohen

**Affiliations:** 1 Autism Research Centre, Department of Psychiatry, University of Cambridge, Douglas House, 18b Trumpington Rd, Cambridge, CB2 8AH, UK

## Abstract

**Background:**

The Autism Spectrum Quotient (AQ) is a self-report questionnaire for quantifying autistic traits. This study tests whether the AQ can differentiate between parents of children with an autism spectrum condition (ASC) and control parents. In this paper, the use of the AQ to define the broader, medium and narrow autism phenotypes (BAP, MAP, NAP) is reported, and the proportion of parents with each phenotype is compared between the two groups.

**Methods:**

A sample of 571 fathers and 1429 mothers of children with an ASC completed the AQ, along with 349 fathers and 658 mothers of developing typically children.

**Results:**

Both mothers and fathers of the diagnosed children scored higher than the control parents on total AQ score and on four out of five of the subscales. Additionally, there were more parents of diagnosed children with a BAP, MAP or NAP.

**Conclusions:**

The AQ provides an efficient method for quantifying where an individual lies along the dimension of autistic traits, and extends the notion of a broader phenotype among first-degree relatives of those with ASC. The AQ is likely to have many applications, including population and clinical screening, and stratification in genetic studies.

## Background

Autism spectrum conditions (ASC) are diagnosed on the basis of behaviour, specifically difficulties in social and communication development, alongside repetitive behaviour and unusually narrow strong interests [1]. The evidence for the genetic basis of ASC initially came from twin studies of classic autism [2,3] and more recently twin studies of autistic traits [4-6]. Progress from these epidemiological findings to identifying specific DNA sequence variations that cause ASC has been slow: replication of results has been hampered by methodological issues such as limited power, varying designs and genotyping, along with imprecise phenotypic definitions [7]. Another reason for limited progress is that although ASC has a high inheritance rate, it is genetically heterogeneous. Rare *de novo *mutations and chromosomal abnormalities could account for as many as 20% of ASC cases, but common allelic variation is also important, suggesting that a categorical approach to case ascertainment may not always be the best approach [8].

Indeed, a case-control or categorical approach to diagnosis ignores the view that autism is not just a spectrum within the clinical population, but that autistic traits are continuously distributed right through the general population [5,9]. Taking seriously the dimensional view necessitates the use of techniques such as quantitative trait loci (QTL) analysis. Quantitative traits are characteristics that can be associated with a particular condition but are also continuously distributed in the non-affected population. If QTL is to be a successful tool in the search for autism susceptibility genes, then an instrument that can quantify autism traits in both affected and non-affected individuals is required. As well as quantifying autism traits within the whole population, such an instrument could also be used to define the broader autism phenotype (BAP).

The BAP is generally considered to be a subclinical set of characteristics or traits that index familiality and/or genetic liability to autism. This conception holds that the BAP is milder but qualitatively similar to the diagnosed autism phenotype. BAP characteristics were first observed by Kanner [10], and were also noted in Folstein and Rutter's early twin study [3], which found a higher concordance rate for a more broadly defined cognitive impairment.

Given the increasing importance of measuring the BAP, not only in genetic studies, but across autism research (for example, in neuroimaging and cognitive studies), it is important to select the right tool. There are already at least four instruments available for the assessment of the BAP: the Family History Interview (FHI), developed by Rutter and Folstein; the Social Responsiveness Scale (SRS) [11]; the Broader Phenotype Autism Symptom Scale (BPASS) [12]; and the Broad Autism Phenotype Questionnaire (BAPQ) [13].

The FHI measures autism-related traits in family members via interview with one member. The instrument covers social and communication skills and range of interests. An algorithm determines the presence or absence of the BAP in the three domains. The FHI was used by Bolton and colleagues [14] to demonstrate higher rates of the BAP in the parents and siblings of probands with autism compared with probands with Down's syndrome. This finding was replicated by Piven and colleagues [15].

The SRS is a 65-item questionnaire, which is completed by an adult informant. Its focus is on the ability of the subject of the questionnaire to engage in emotionally appropriate reciprocal social interaction and communication. Using the SRS, a higher number of autistic traits have been observed in the siblings of children with autism [16].

The BPASS was developed specifically for use in QTL studies [12]. This instrument comprises seven semistructured interview items and six observation items, which together cover social motivation, social expressiveness, conversational skills and repetitive/restricted behaviours. The BPASS is for use with both adults and children.

The most recently published instrument, the BAPQ, comprises 36 items, 12 in each of three subscales: aloof personality, pragmatic language deficits, and rigid personality. Ideally, scores from self- and informant reports are combined to give a best-estimate rating. The BAPQ has been validated using direct clinical assessment of BAP [13].

We suggest that when choosing an instrument to measure autistic traits in both the affected and non-affected populations, the following qualities are important. (i) A good BAP measure should be quantitative, with a wide range of possible scores, avoiding both ceiling and floor effects. (ii) The content validity should be such that the instrument can distinguish people with from those without an ASC. (iii) Versions of the same instrument should be available for use with individuals of different ages, both adults and children. (iv) Ideally, the instrument should produce a normal or near-normal distribution in the general population. (v) The instrument should have good test-retest reliability. (vi) It should have good cross-cultural applicability. (vii) It should correlate with either biological and/or psychological measures of the broader phenotype (concurrent validity). Finally, (viii) it should be quick and easy to use: participants in research projects should be subjected to the least amount of testing possible, without compromising the validity of the research project.

Bearing in mind these qualities, each of the four instruments described above have some weaknesses: the FHI and BPASS are not truly quantitative and are both time-consuming to administer, the SRS is not normally distributed, and the BAPQ is currently limited to adults.

In this paper, we propose that an alternative to the four instruments already available is the Autism Spectrum Quotient (AQ) [9]. The AQ was developed to assess where an individual lies on the autism spectrum (that is, how many autistic traits that person exhibits). Originally designed for adults, there are now child ( 4-11 years old) [17] and adolescent (12-16 years old) [18] versions available (both completed by parents to improve accuracy). The questionnaires are freely available to download http://www.autismresearchcentre.com.

The AQ for adults has the format of a self-report, forced-choice questionnaire and is at the reading level of a typical 10-year-oldl. It can be used by adults with an IQ in the average range, who can read and understand at least to this level. There are 50 items, covering behaviours across five domains: communication, social skills, attention switching, imagination, and attention to detail. Each item has the format of a statement with which the respondent rates how strongly they agree or disagree, using a four-point scale. In fact, each item is only scored as '1' if the person reports the autistic trait, and '0' if they do not, so total scores on the AQ can range from 0 to 50. Some researchers have used a four-point Likert scale for the AQ [19-21], but this does not significantly alter the pattern of results. The AQ is quick and easy to use and produces a near-normal distribution in the general population [9].

The adult AQ has been used extensively, and has been shown to have consistent results both across time [22] and culture [20,23]. The AQ demonstrates high heritability [5]. The AQ score is a good predictor of clinical diagnosis [24], correlates with brain function [25], single nucleotide polymorphisms (SNPs) in candidate genes [26], social attention [27,28] and even prenatal testosterone levels [29].

A previous study, by Dorothy Bishop and colleagues, suggested that AQ scores differentiate parents of children with an ASC from control parents on two subdomains: communication and social skills [29]. Although this was a pioneering study, its limitation was that it only tested 65 mothers and 46 fathers of children with a pervasive developmental disorder (PDD) diagnosis, and 48 mothers and 37 fathers of control children. Although this study indicates the potential of the AQ to serve as a measure of the BAP, it is not clear whether the lack of significance on three of the five subdomains of the AQ is because these play no role in the BAP or because the sample size was too small. Therefore, in the current study, we used the AQ with a much larger sample of parents.

Our first aim was thus to test if the results found by Bishop and colleagues [29]could be replicated in a new, and considerably larger, sample. The second aim was to test the proportion of parents of children with an ASC who demonstrate the BAP, the medium autism phenotype (MAP) and the narrow autism phenotype (NAP). The MAP and NAP are new concepts. An individual with the NAP has a large number of autistic traits, and most (but not all) people with the NAP will also have a diagnosis on the autism spectrum. Not all individuals with the NAP will be diagnosed because there are many reasons why an individual may seek or receive a diagnosis: the NAP is one band within the phenotypic spectrum and does not take into consideration the individual's context, which may mediate whether they actually need or receive a diagnosis. Using the AQ, we define the NAP as those scoring ≥ 3 standard deviations (SDs) above the mean. An individual with the MAP has a medium number of autistic traits (defined as individuals scoring between 2 to 3 SDs above the mean on the AQ), and an individual with the BAP has even fewer traits, but still significantly more than average (defined as individuals scoring 1 to 2 SDs above the mean on the AQ). People with the MAP or BAP are unlikely to require clinical intervention, but their phenotypic status may be informative at a genetic level.

## Methods

### Instrument

Full details about the construction of the AQ are available elsewhere [9]. The AQ consists of a series of 50 statements. Participants are asked to indicate whether they 'strongly agree', 'slightly agree', 'slightly disagree' or 'strongly disagree' with each statement. Each item scores zero or one, with one point being awarded if the participant chooses the 'autistic trait' response. On half the items, the 'autistic trait' response is 'slightly/strongly agree', and on half the items the 'autistic trait' response is 'slightly/strongly disagree'. A total score is calculated by summing across items. The BAP, MAP and NAP scores are calculated using the mean and SD from the combined male and female control individuals shown in Table 1 (n = 1761, mean ± SD = 16.3 ± 5.9; taken from [22]) BAP is defined as AQ scores of 1 to 2 SDs above the mean (AQ scores of 23 to 28). MAP is defined as AQ scores of 2 to 3 SDs above the mean (AQ scores of 29 to 34). NAP is defined as AQ scores ≥ 3 SDs above the mean (AQ scores of 35+).

**Table 1:** Number of children developing typically in the 1582 ASC families

Number of siblings developing typically	0	1	2	3	4	5
Number of families	1260	217	82	14	8	1

ASC = autism spectrum condition.

### Participants and procedure

Two groups of parents took part in the study: parents of children with a diagnosis on the autism spectrum and parents of families who only had children who were developing typically. The parents of children with an ASC were recruited from families who registered on the Cambridge University Autism Research Centre volunteer database between 2002 and 2009 (online at http://www.autismresearchcentre.com). Ethics approval for the database and questionnaire collection was provided by the Cambridge Psychology Research Ethics Committee. Parents gave informed consent to take part in the study electronically.

Parents registering on the database are asked to state the diagnosis of their child, who made the diagnosis, and where and when. In addition, parents are asked to provide information about the IQ and language development of their child, although this is not obligatory. After registering, the parents are invited to complete an AQ, and are asked to encourage the other biological parent to also register and complete an AQ. The advantage of using an online website to collect these data is that large samples can be collected. The disadvantage is that diagnosis cannot be validated in every case. All parents included in this study reported that their children had received their diagnosis from experienced clinicians in recognized clinics and according to the criteria from the *Diagnostic and Statistical Manual of Mental Disorders*, 4th edition (DSM-IV) or the *International Statistical Classification of Diseases and Related Health Problems*, 10th Revision (ICD-10).

In total, 1582 families took part, with 571 fathers and 1429 mothers completing the AQ. There were questionnaires from 418 couples. The mean age of the fathers for whom these data were available (n = 551) was 44.0 ± 6.9 years and for mothers (n = 1228) was 41.2 ± 7.5 years. The numbers of diagnosed and developing typically children in the 1582 families are shown in Tables 1 and 2. There were 1752 children with a diagnosis of ASC:1472 male and 280 female, giving a male:female ratio of 5.3:1. of he 1752 children, 727 were reported to have autism, 725 to have Asperger's syndrome (AS), 185 to have high functioning autism (HFA) and 115 to have pervasive development disorder. As a further check on diagnostic subtype, the registration process asks about comorbid language delays or learning difficulties. A diagnosis of AS is only registered if the parent indicates that the child did not have any language or general developmental delay. A diagnosis of HFA is only registered if the parent indicates that there was no general developmental delay even if language was delayed.

**Table 2:** Number of children with a diagnosis in the 1582 ASC families

Number of diagnosed children	1	2	3	4
Number of families	1423	150	7	2

ASC = autism spectrum condition.

For the control group, the AQ was sent to the parents of 1255 children (633 girls, 622 boys) who were participating in a large epidemiological study of social and communication skills in primary school-age children [30-32]. This part of the project was ethically approved by the National Health Service Suffolk Research Ethics Committee, and written informed consent was obtained from participating parents. Two copies of the questionnaire and two consent forms were posted to each family: one set for the mother and one set for the father. A post-paid envelope was included for participants to return the questionnaires and consent forms. This sample was originally ascertained by inviting 136 mainstream primary schools in Cambridge City, east and south Cambridgeshire, and Fenland in the UK to participate in research (n = 92 (68%), agreed). In total, 1012 questionnaires were received from 669 families (661 from mothers, 351 from fathers) with 343 pairs, a 40% response rate. Two families were excluded because they reported a child with a diagnosis or suspected diagnosis on the autism spectrum. One family was excluded because they reported a child with Down's syndrome. Questionnaires from 666 families (558 mothers and 349 fathers) with 341 pairs were analysed. The mean age (at the time of completing the AQ) of the fathers for whom these data were available (n = 344) was 44.6 ± 5.0 years and for mothers (n = 644) was 42.4 ± 4.7. The children in these families were, according to parental report, all developing typically, and the numbers are shown in Table 3; in total, there were 1532 children in these families.

**Table 3:** Number of children in the 666 control families

Number of children	1	2	3	4	5	6	7
Number of families	85	351	183	41	5	0	1

## Results

**Figure 1:**
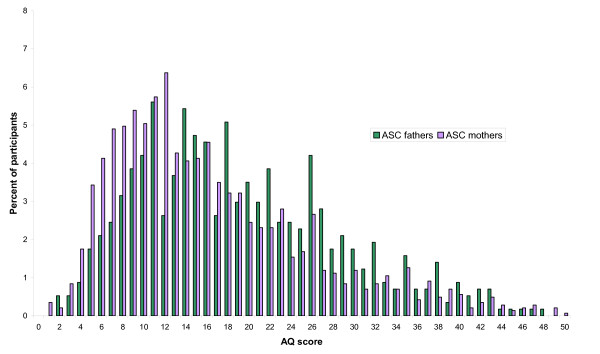
**Autism Spectrum Quotient (AQ) scores in the Autism Spectrum Condition (ASC) parent group**.

**Figure 2:**
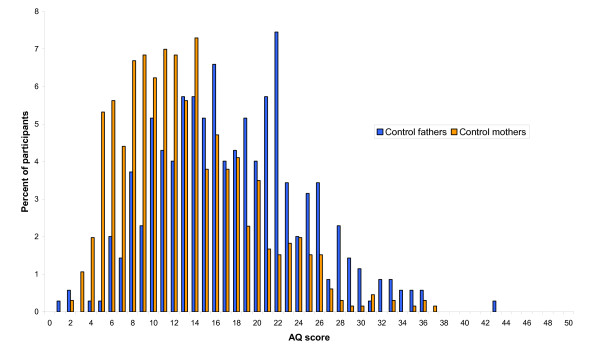
**Autism Spectrum Quotient (AQ) scores in the control parent group**.

The mean AQ scores and standard deviations are shown for the two groups in Table 4, along with control data and AS data [22] for comparison. The distribution of scores in the two parent groups are shown in Figure 1 and Figure 2.

**Table 4:** Mean total AQ scores for the parent groups

	Males			Females		
		
	n	Mean	SD	n	Mean	SD
ASC parents	571	19.2	9.5	1429	16.4	9.5

Control parents	349	17.7	6.9	658	13.1	6.3

AS group	69	35.9	7.8	56	37.4	8.2

Control individuals	723	17.4	6.2	1038	15.5	5.6

Data from an AS group and controls, taken from [22] are also shown for reference.

AQ = Autism Spectrum Quotient; ASC = autism spectrum condition; AS = Asperger's syndrome.

Analysis of variance (ANOVA), with between-subject factors of 'group' (ASC parents versus control parents) and 'gender' was carried out on total AQ score. There was a significant main effect of group (*F*_(1,3003) _= 45.8, *P *< 0.001), with the ASC parents scoring higher than the control parents, and of gender (*F*_(1,3003)_) = 104.3, *P *< 0.001), with males scoring higher than females. Although the group × gender interaction was significant (*F*_(1,3003) _= 6.0, *P *< 0.05), simple effect tests indicated that the gender difference held up in both groups separately (ASC parents: *F*_(1,3003) _= 42.0, *P *< 0.001; control parents: *FF*_(1,3003) _= 62.5, *P *< 0.001) and that the group difference also held up when each gender was tested separately (males: *F*_(1,3003) _= 6.9, *P *< 0.01; females: *F*_(1,3003)_) = 65.4, *P *< 0.001). The effect sizes for the group, gender, and group × gender interaction were all small: partial eta squared (η^2^) values were 0.02, 0.03 and 0.002 respectively, indicating that these factors accounted for just a small amount of the overall variance.

Table 5 shows the means and standard deviations of the five subdomains of the AQ in the parent groups only. Also shown are the F values and partial η^2 ^values generated by separate ANOVAs, with between-subject factors of group (ASC parents versus control parents) and gender, for each of the five subdomains. Inspection of the means suggests that for each subcategory, males scored higher than females, and the ASC parents scored higher than the control parents. The exception to this was the subcategory 'attention to details'; in this, there was no sex difference and the control parents scored higher than the ASC parents. These observations were partly confirmed by the ANOVAs and simple effect tests. Similar to the total AQ score, the subcategories 'attention switching' and 'imagination' had significant main effects of group and gender and a significant group × gender interaction. Also similar to the total AQ score, simple effect tests indicated that for both subcategories the sex difference held up in both groups separately, and that the group difference also held up when each sex was tested separately (*P *< 0.01 for all). The category 'communication and social skills' both had significant main effects of group and gender, but no interaction between them, whereas the subcategory 'attention to detail' only had a significant main effect of group. Again, all the effect sizes are small.

**Table 5:** Mean (and standard deviation) scores on each of the AQ subcategories in the parent groups

	Communication	Social skills	Attention switching	Imagination	Attention to detail
**ASC fathers**	3.1 (2.4)	4.0 (2.9)	4.5 (2.6)	3.2 (2.3)	4.4 (2.3)

**ASC mothers**	2.6 (2.4)	3.1 (2.8)	4.0 (2.5)	2.4 (2.2)	4.4 (2.3)

**Control fathers**	2.6 (1.9)	3.1 (2.5)	4.2 (2.2)	3.1 (2.1)	4.7 (2.1)

**Control mothers**	1.7 (1.6)	1.9 (2.1)	3.1 (2.0)	1.8 (1.7)	4.7 (2.2)

**F value for group main effect (and partial eta^2^)**	55.6**	91.8**	39.4**	18.4**	11.1*
	(0.02)	(0.03)	(0.01)	(0.006)	(0.004)

**F value for sex main effect (and partial eta^2^)**	58.6**	85.9**	71.0**	156.0**	0.01
	(0.02)	(0.03)	(0.02)	(0.05)	(0.00)

**F value for group x sex interaction (and partial eta^2^)**	3.6	2.1	9.2*	6.9 *	0.01
	(0.001)	(0.001)	(0.003)	(0.002)	(0.00)

F values (and partial eta^2^) from the ANOVAs are presented.

*P < 0.01, ** *P *< 0.001

AQ, Autism Spectrum Quotient; ASC, autism spectrum condition

The number of parents matching each of the described phenotypes (the BAP, MAP and NAP) was computed along with the number scoring in the average range (within 1 SD of the mean) and the low range (< 1 SD below the mean). These data are presented in Figure 2.

**Figure 3:**
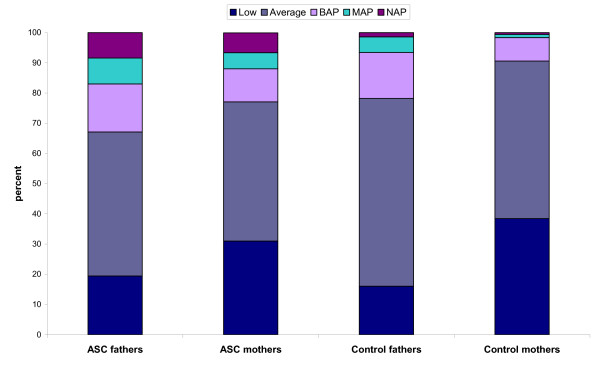
**Percent age of parents with each phenotype**. ASC = autism spectrum condition; BAP = broader autism phenotype; MAP = medium autism phenotype; NAP = narrow autism phenotype

Examination of Figure 3 shows that ASC fathers had the highest percentage of scorers across the BAP, MAP and NAP range (approximately 33%), compared with 23% of ASC mothers, 22% of control fathers and 9% of control mothers. The finding that a similar proportion of ASC mothers and control fathers scored in the BAP or higher range is consistent with the 'extreme male brain' theory of autism [33]. More ASC mothers scored in the MAP and NAP range than control fathers (χ^2 ^= 8.2, degree of freedom (d.f.) = 1, *P *< 0.01). Control mothers had the lowest proportion in the BAP, MAP and NAP ranges, and the fewest number scoring in at least the BAP range. About 50% of the ASC parents and control mothers score in the average range, compared with about 60% of the control fathers. Control mothers were over-represented in the low range compared with ASC mothers (χ^2 ^= 11.2, d.f. = 1, *P *< 0.01). There was no difference between the two groups in the proportion of fathers scoring in the low range (χ^2 ^= 1.7, d.f. = 1, *P *> 0.05).

## Discussion

The AQ was developed almost 10 years ago as an instrument to rapidly quantify autistic traits in both clinical and non-clinical samples. Evidence for the construct validity of the AQ and the use of a self-report instrument for quantifying autistic traits comes from a wide variety of autism-related studies in which AQ score correlates with outcome [24-26,34-42]. The AQ is thus a reasonable tool for assessing whether individuals can be classified as having the BAP. Measuring the BAP is increasingly important in genetic studies, and is also important in describing participants in other types of research. Although it may seem contradictory to be employing cut-off points on a scale that was devised precisely to avoid categorical distinctions, other psychometric measures (such as IQ) have long used this approach because there may be predictive value in subgrouping by severity. We thus also introduce the terms MAP and NAP as additional subgroup classifications, while acknowledging that the cut-off points are purely statistically defined (by number of SDs above the mean) rather than 'carving Nature at its joints'.

In the present sample, parents of children with ASC scored significantly higher on the AQ than control parents, both on total AQ score and on four of five of the subcategories. This replicates and extends the earlier finding by Bishop and colleagues [29] in a much larger sample.

The effect sizes were all small, which is not surprising, as we would not predict the BAP to be present in all parents. Comparing the differences between parents who do and do not show the BAP will be important in future research. Furthermore, our sample is heterogeneous; it includes parents of children with different diagnoses on the autism spectrum, some of whom come from multiplex and some from simplex families. In addition, we found not only are the rates of BAP higher in parents of children with an ASC than in control parents, but the rates of MAP and NAP are also higher, as measured by the AQ. This was true for both mothers and fathers.

### Study limitations

There are three methodological limitations with this study. First, information about the families, including child diagnosis, was fully reliant on parental report. Secondly, the questionnaires in the ASC and control groups were collected in slightly different ways. The questionnaires from the ASC group were collected from parents registering an interest in taking part in research, mostly via an online website, although some were paper copies. The questionnaires were collected over a period of 7 years. By contrast, the control group questionnaires were collected by post, over a period of a few weeks. Although we do not think a person's AQ score will be affected by the method of questionnaire administration (because the wording remains identical) this assumption warrants future testing. The time difference between data collection was not ideal, because public awareness of autism and related conditions has increased. However, it is not clear what influence, if any, this would have on individual's response. Future studies should try to ensure that data collection methods are identical for all participants.

Finally, a third limitation is that our control group only comprised families with children who were developing typically. Future studies could also include families who have children with a different developmental disability, to test if there are any effects on how parents report their own characteristics after the diagnosis of a child is made. We doubt this would have contributed to the present results, because the AQ measures a range of domains, including both social and non-social (attentional) characteristics.

## Conclusions

This study provides evidence that the AQ is a useful tool for assessing autism phenotypes in non-clinical samples. The novel terms 'medium autism phenotype' and 'narrow autism phenotype' are introduced to extend the established term 'broader autism phenotype', and precise methods of measuring each are provided. We found that 33% of fathers of children with ASC and 23% of mothers scored at or above the BAP cut-off point, with significantly more parents of children with ASC showing MAP and NAP than was seen among control parents. Whether the broad, medium and narrow phenotypes are mirrored by either neural, cognitive, endocrine, proteomic or genetic differences will be of interest to test in the future.

## Competing interests

The authors declare that they have no competing interests.

## Authors' contributions

All authors read and approved the final manuscript. SW and SBC conceived the study and participated in its design. SW collated the ASC data, performed the statistical analysis and drafted the manuscript. BA and CA collected the control data.
